# Managing abdominal cocoon syndrome complicated by intestinal necrosis and unexpected amelioration of depression after surgery: a case report

**DOI:** 10.1186/s13256-024-04650-9

**Published:** 2024-07-06

**Authors:** Judong Zhang, Yifang Hsieh, Kunming Zheng, Jing Xu

**Affiliations:** 1 Department of General Surgery, Tianjin Union Medical Center, The Affiliated Hospital of Nankai University, Tianjin, 300121 China; 2 Tianjin Key Laboratory of General Surgery in Construction, Tianjin Union Medical Center, Tianjin, 300121 China; 3 School of Medicine, Nankai University, Tianjin, 300071 China

**Keywords:** Abdominal cocoon, Case report, Depression, Encapsulating sclerosing peritonitis, Small bowel obstruction

## Abstract

**Background:**

Abdominal cocoon is a very uncommon yet dangerous cause of intestinal obstruction.

**Case presentation:**

We present a case of a 62-year-old Asian male patient with a history of depression who exhibited an idiopathic abdominal cocoon complicated by necrosis. Upon laparotomy investigation, nearly the entire small intestine was enveloped in a thick membrane resembling a cocoon, and it was discovered that he lacked a greater omentum. The patient recovered well and was discharged on an oral diet on the 20th day following surgery. During the 3-month follow-up, the patient was asymptomatic, even gaining 10 kg in weight, and noted that his depression had improved.

**Conclusions:**

Small bowel obstruction presents with nonspecific symptoms, posing challenges in differential diagnosis. Contrast-enhanced computed tomography is recommended since it facilitates precise preoperative assessment, optimizing surgical planning and reducing postoperative complications. Remarkably, cessation of antidepressant medication post-surgery hints at a potential correlation between omental deficit, gut microbiota alterations, and depressive symptoms.

## Introduction

Abdominal cocoon (AC), sometimes referred to as encapsulating sclerosing peritonitis (ESP), is a very uncommon yet dangerous cause of intestinal obstruction. It is distinguished by the existence of fibrous membranes that encircle the intestines, creating a structure similar to a butterfly cocoon that may interfere with the peritoneal membrane’s regular physiological function. Although the prevalence of AC is unknown, it ranges from 1.4% to 7.3% among peritoneal dialysis patients [1]. According to Machado *et al.*, 72% stomach discomfort, 44% abdominal distension, 30% abdominal mass, and nausea/vomiting can be observed among typical patients [2]. The combination of radiological signatures from computed tomography and the clinical pathognomonic feature is best described for preoperative diagnosis and appropriate management of AC [3]. Despite the high incidence of adhesive intestine and early postoperative small bowel obstruction (EPSBO), which typically leads to a postoperative recurrence of obstruction, surgery remains the best option for patients with AC [4]. This work has been reported in line with the CARE criteria [5].

## Case report

**Fig. 1:**
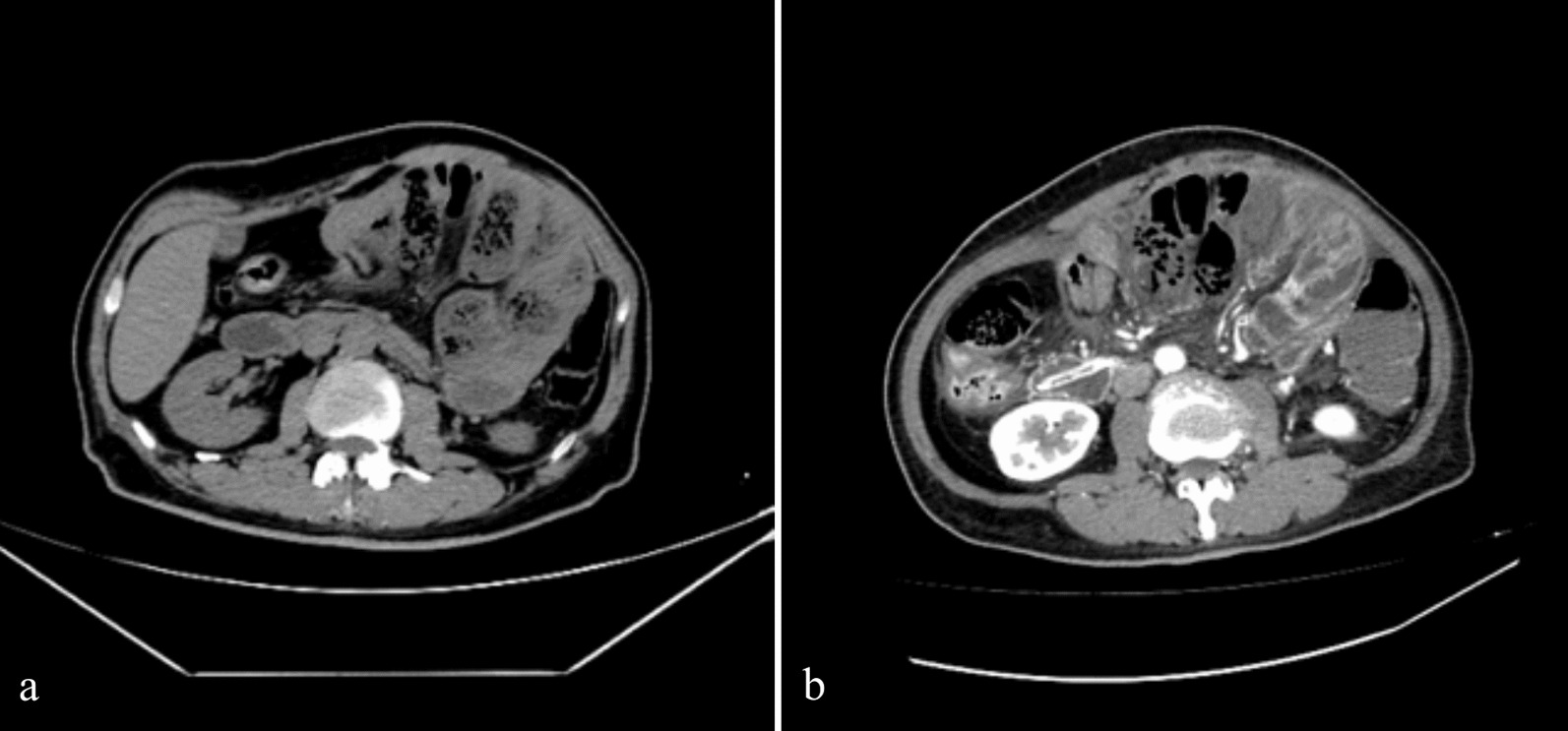
Computed tomography (CT) versus contrast-enhanced computed tomography. **a** Small intestine irregularly distributed and gathered in the left middle quadrant of abdomen indicating possible intraabdominal hernia; **b** contrast-enhanced CT scan obtained through the middle abdomen showing a conglomerate of multiple twisting and deformation intestinal loops surrounded by a enhancing thick sac-like structure

A 62-year-old Asian male presented to us with left upper quadrant abdomen pain and radiating pain in the lower back for 1 day after consuming a hard solid food. The discomfort was constant, mild to moderate, and accompanied by nausea and vomiting. Acute intestinal obstruction was revealed on computed tomography (CT) (Fig. 1a). He had no prior medical history of trauma, surgery, altered bowel habits, abdominal pain, or tuberculosis. Upon closer inspection, he appeared ill and distressed. His vitals were as follows: blood pressure of 130/80 mm Hg, respiration rate of 20 breaths per minute, and pulse of 85 beats per minute (regular). He did not have a fever. A generalized abdominal tenderness was observed without guarding and rigidity over the central lower/left upper part of the abdomen and a 15-by-20-cm palpable mass anterior to the left of the umbilicus. No gut sounds were audible. Laboratory investigations showed a white blood cell count (WBC) of 14.49 × 10^9^/L, neutrophil percentage (N%) of 89.2%, and C-reactive protein (CRP) of 60.6 mg/L. Following the insertion of a long intestinal tube into the Tretiz’s ligament, the patient’s distension and abdominal pain were eased. The patient experienced a rapid onset of high fever and chills after receiving treatment for 2 days (Temperature: 39.3 degrees Celsius). Left upper quadrant discomfort was clearly visible throughout the abdominal examination, and there was no rebound pain or high muscle tone. A contrast-enhanced computed tomography (CECT) scan revealed intestinal obstruction, necrosis, and characteristics of an abdominal cocoon (Fig. 1b). Laparotomy was chosen on the basis of clinical and radiographic characteristics.

**Fig. 2:**
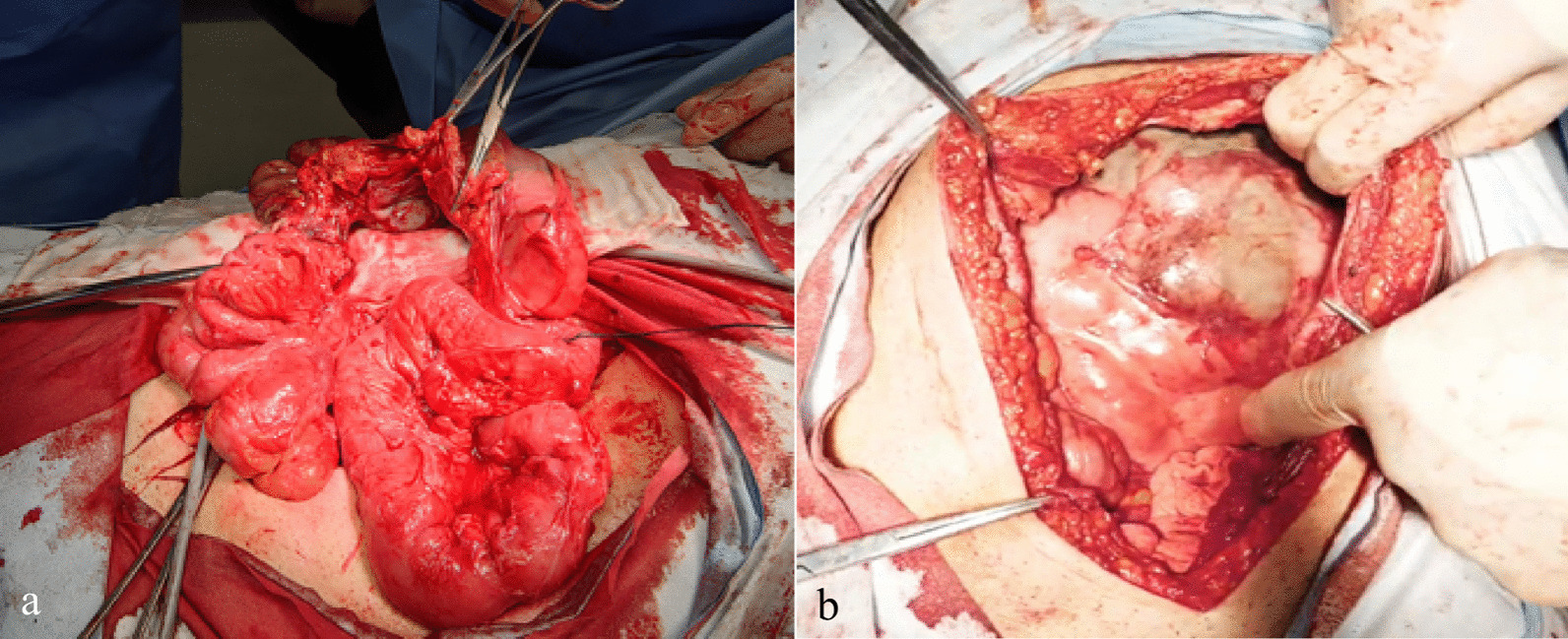
Intraoperative small bowel outlook. **a** Thicker fibrous capsules encasing nearly the whole of the hypoperfused, tiny, viable small bowel suggestive of an abdominal cocoon along with a 50-cm blackened and necrotic small intestine loop; **b** absence of greater omentum

**Fig. 3:**
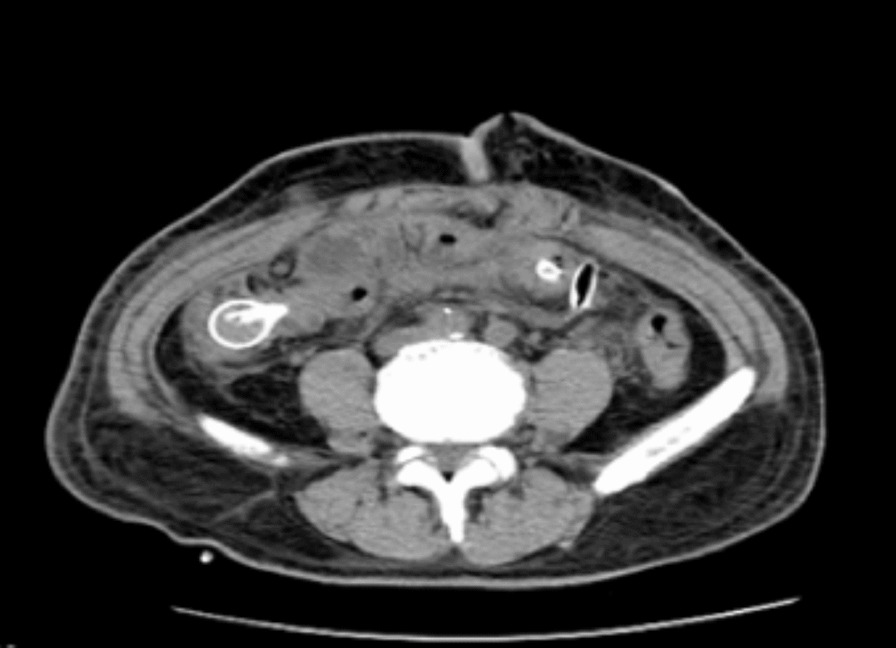
Abdomen computed tomography (CT) scan after surgery. The prior long intestinal tube was sent to the cecum and also used to release the entrapped residual small intestine

**Fig. 4:**
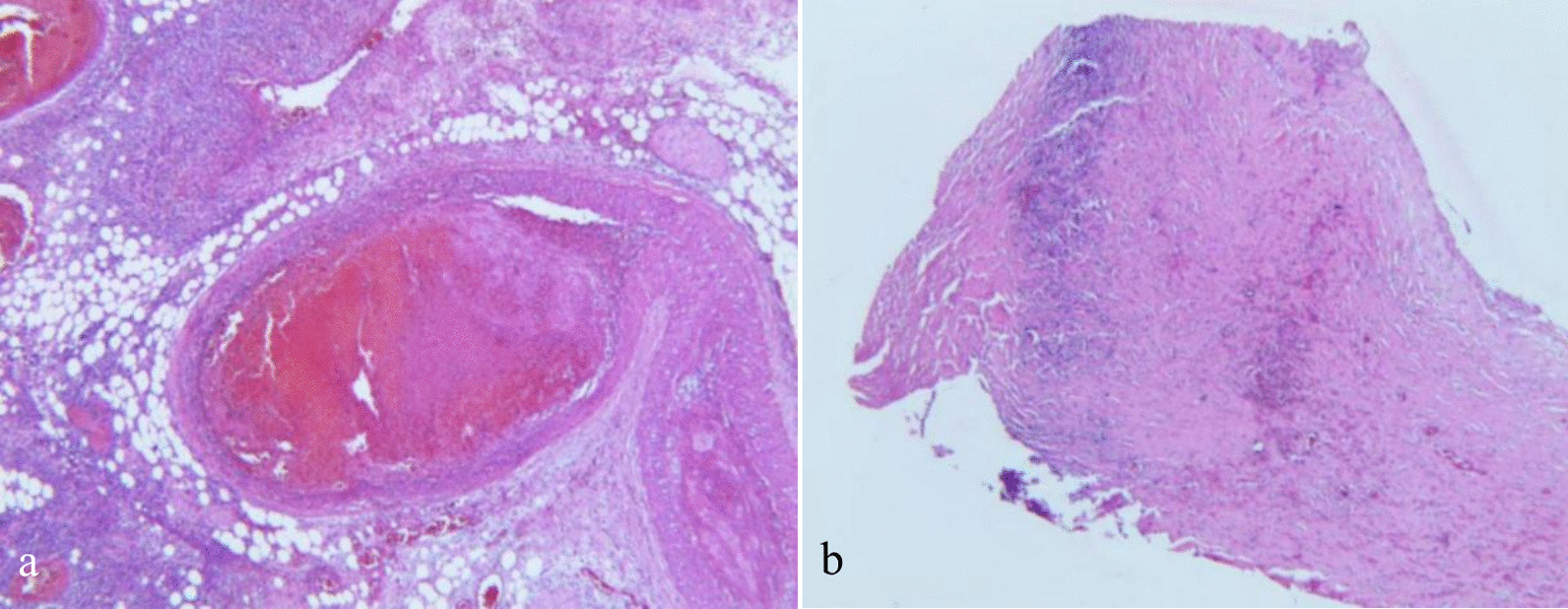
Histopathological findings. **a** Focal vascular thrombosis, congested blood vessels, and proliferating fibrosis tissue; **b** histologic examination of the membrane tissues showing proliferation of inflammatory cells

Intraoperative observations revealed thicker fibrous capsules encasing nearly the whole of the hypoperfused, tiny, viable small bowel suggestive of abdominal cocoon, with dense inter-loop membranous adhesion along with a 50-cm blackened and necrotic small intestine loop (Fig. 2a) about 70 cm proximal to the ileocecal junction. No greater omentum could be detected (Fig. 2b). As a result of these findings, the patient underwent an incision made in thick fibrous membrane as well as the necrosis intestine loop with adhesiolysis and manual end-to-end small intestinal anastomosis. We sent the prior long intestinal tube to the cecum and released the entrapped residual small intestine (Fig. 3). Histopathological examination of the small bowel revealed focal vascular thrombosis, congested blood vessels, and proliferating fibrosis tissue (Fig. 4a). There was no indication of malignancy or atypia, and features of inflammation with mild inflammation, including extensive diffuse neutrophil infiltration with dilated lymphatics, were observed, whereas the membrane revealed a few indications of cell inflammation (Fig. 4b). Overall, the histological and intraoperative results proved that the patient had idiopathic sclerosing peritonitis.

**Fig. 5:**
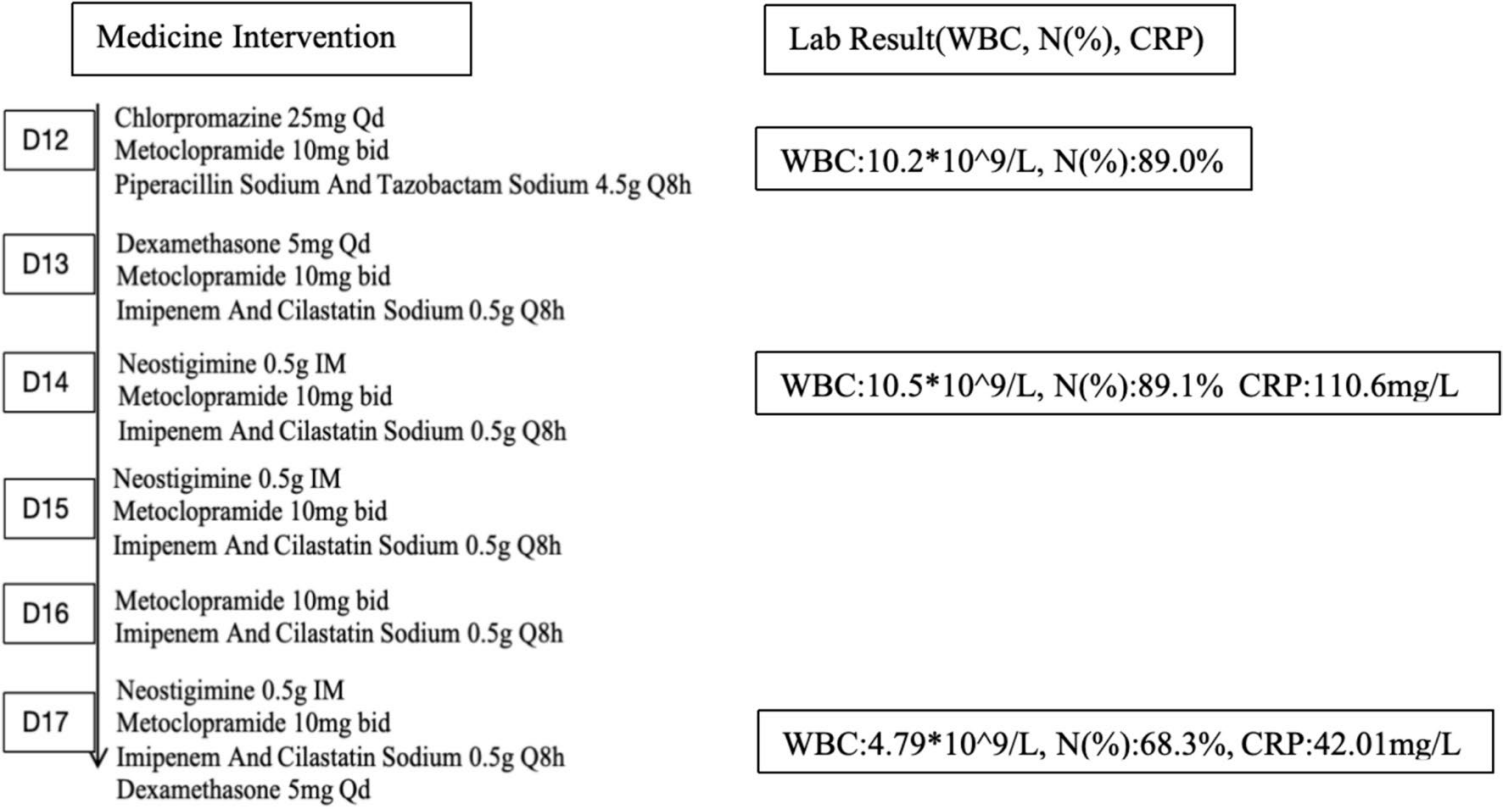
Treatment after day 12 and the changes in the lab results. Normal range: white blood cell count (WBC) 3.5–9.5 × 10^9^/L, neutrophil percentage (N%) 40–75%, C-reactive protein (CRP) 0–10 mg/L

Postoperatively, the patient was given permission to be discharged on the 20th day, showing no symptoms of obstruction in their bowel movements and the capacity to tolerate a regular diet. We encouraged the patient to have an early ambulation at day 5 following surgery, and the stomach and intestinal peristalsis recovered. The patient started to consume a clear liquid diet and a semi-liquid diet at day 7 and day 9, respectively. The patient experienced a fever with a peak body temperature of 38.6 °C, along with decreased exhaustion, feces, and light gut sounds. WBC of 10.02 × 10^9^/L and N% of 89% was shown by the routine blood work, suggesting that there may be an inflammatory intestinal blockage. Following proper treatment (Fig. 5), the patient recovered well and was discharged with the long tube retained 20 days following the surgery. During the 3-month follow-up, the patient was asymptomatic, even gaining 10 kg in weight, and noted that his depression had improved.

## Discussion

The term abdominal cocoon describes an uncommon disorder in which the small bowel is completely or partially encased in a fibrocollagenous sac. There are two types of AC: the idiopathic form and the secondary form. As mentioned above, though a number of theories have been put forth, including retrograde menstruation combined with a viral infection, retrograde peritonitis, cell-mediated immune tissue damage triggered by a gynecologic infection, and intrinsic abnormalities of the membranous greater omentum, which may descend along the transverse colon and encase the digestive tract, the etiology of the idiopathic form is still unknown [6, 7]. Peritoneal irritation can also lead to inflammation, which can then trigger peritoneal mesothelial cell proliferation and hyperplasia, as well as peritoneal capillary angiogenesis, which is assumed to be the source of “cocoon” production [8]. Owing to the lack of a greater omentum in this situation, we tend to interpret the cocoon formation as the greater omentum encasing the intestine. Furthermore, as the patient indicated, the palpable bulge in the abdomen has been present for more than 20 years. The AC etiology in this case could be a congenital defect, and the consumption of hard solid foods could have prompted the symptom.

Small bowel obstruction can be caused by various diseases, which means that the clinical manifestation of AC, specifically in this case, is not specific, thus making it hard to obtain a differential diagnosis. Ultrasound, CT, and plain abdominal X-rays are a few imaging modalities that can help in the diagnosis of AC [9–11]. We strongly advocate the use of CECT since the arterial phase allows us to establish a more precise preoperative diagnosis as well as select the best surgical intervention approach to minimize the likelihood of postoperative complications, thus shedding light on the possible diagnosis procedure.

In this case, the diagnosis of type I primary abdominal cocoon resulted from characteristics suggestive of AC combined with the intraoperative discovery of a cocoon membrane encasing nearly the whole small bowel in the absence of any secondary etiology [12]. Even though necrosis necessitated intestinal resection, potential early postoperative obstructions and adhesions were avoided via a long tube to release the entrapped intestine rather than pealing off the small bowel’s fibrosis membrane. Moreover, the long tube can be used to assist with postoperative food consuming. Furthermore, by employing manual suturing as opposed to staplers, residual intestinal damage can be lessened and sufficient length of the small intestine can be preserved, lowering the likelihood that short bowel syndrome will manifest. The average duration of postoperative stay and oral intake for patients who underwent intestinal stenting was 20 and 14 days, respectively, and their EPSBO rate was significantly lower than that of patients who did not undergo intestinal stenting [4]. Our patient was started on oral intake at day 7 after surgery; though he had experienced high fever and suspected inflammatory bowel obstruction at day 12 after surgery, the symptoms quickly subsided after giving proper treatment. We believe that early ambulation and the use of the long tube aided in the recovery of bowel function. Although it was difficult to prevent postoperative complications, we made every effort to limit the harm, which helped to ensure an uneventful outcome, allowing for discharge 20 days following the procedure.

The patient has a 2-year history of depression and takes antidepressants on a regular basis. The connection between depression and microbial dysbiosis has long been recognized, and the pathophysiology of depression has been linked to increased bacterial translocation brought on by the gastrointestinal tract’s impaired tight junctions and barrier integrity [13]. Additionally, antidepressants also seem to play a role in microbial dysbiosis [14]. It is likely that the abdominal cocoon resulted in aberrant junctions and a low-grade inflammatory milieu and changed the composition of the gut bacteria, which in turn caused depression. When the AC was removed, the inflammatory condition decreased, the gut microbiota began to shift, and, eventually, the depression improved and the patient no longer required antidepressants.

## Conclusion

Abdominal cocoon is a rare cause of small bowel obstruction. Though preoperative diagnosis is usually difficult in AC owing to the nonspecific clinical manifestation, usage of CECT is highly recommend. In this case, despite having a partial bowel resection, the patient recovered well and was asymptomatic 3 months after the procedure. The novel strategy of releasing the entrapped bowel by using a long tube as well as choosing a manual suture rather than a stapler may have had an effect on the reduced rate of postoperative problems. Unexpectedly, the patient’s depression had ameliorated after surgery, suggesting a potential interplay between innate greater omentum deficit, altered gut microbiota, and chronic low-grade inflammation state of the gut that contributed to the development of depression.

## Data Availability

The datasets used and analyzed during the current study are available from the corresponding author on reasonable request.

## Abbreviations

AC: Abdominal cocoon
CECT: Contrast-enhanced computed tomography
ESP: Encapsulating sclerosing peritonitis
CT: Computed tomography
EPSBO: Early postoperative small bowel obstruction
